# Fragmentation trees for the structural characterisation of metabolites

**DOI:** 10.1002/rcm.6340

**Published:** 2012-08-31

**Authors:** Piotr T Kasper, Miguel Rojas-Chertó, Robert Mistrik, Theo Reijmers, Thomas Hankemeier, Rob J Vreeken

**Affiliations:** 1 Netherlands Metabolomics CentreEinsteinweg 55, Leiden, The Netherlands; 2 Leiden/Amsterdam Centre for Drug Research (LACDR), Leiden UniversityEinsteinweg 55, Leiden, The Netherlands; 3 HighChem. Ltd.Bratislava, Slovakia

## Abstract

Metabolite identification plays a crucial role in the interpretation of metabolomics research results. Due to its sensitivity and widespread implementation, a favourite analytical method used in metabolomics is electrospray mass spectrometry. In this paper, we demonstrate our results in attempting to incorporate the potentials of multistage mass spectrometry into the metabolite identification routine. New software tools were developed and implemented which facilitate the analysis of multistage mass spectra and allow for efficient removal of spectral artefacts. The pre-processed fragmentation patterns are saved as fragmentation trees. Fragmentation trees are characteristic of molecular structure. We demonstrate the reproducibility and robustness of the acquisition of such trees on a model compound. The specificity of fragmentation trees allows for distinguishing structural isomers, as shown on a pair of isomeric prostaglandins. This approach to the analysis of the multistage mass spectral characterisation of compounds is an important step towards formulating a generic metabolite identification method. Copyright © 2012 John Wiley & Sons, Ltd.

One of the central tasks of metabolomics is to identify metabolites in complex biological mixtures and to decode their structure. This is a challenging but essential task, because unless the identity of the studied metabolite is known, its quantitative data cannot be related to its biochemical role. This requires further developing and optimising the available analytical techniques in order to yield a robust metabolite identification platform.

Nuclear magnetic resonance (NMR)^[^^1^^,^^2^^]^ and mass spectrometry (MS)^[^^3^^]^ are the methods most commonly used for the structural characterisation of chemical compounds. NMR offers a rapid and detailed analysis of the structure of the (un)known compound but the technique is severely limited due to its relatively low sensitivity. MS, on the other hand, offers high sensitivity and specificity^[^^4^^]^ resulting in elemental formulas.^[^^5^^]^ However, discerning between (positional) isomers remains a challenge, even if the core structure of the molecule is known. Furthermore, in specific, fortunately rare, cases simply obtaining a protonated or deprotonated molecule can be a challenge as well. In the latter case, a more targeted approach is required to elucidate the structures of these compounds.

Obviously, an elemental formula is not specific enough to identify a metabolite. Its structure can be further characterised by gas-phase fragmentation reactions, e.g. collision-induced dissociation (CID). The resulting fragmentation spectrum reflects the structure of the precursor ion: the masses of the obtained product ions and their relative abundances characterise the structure of the precursor ion and the experimental fragmentation conditions. In this way, a fragmentation spectrum offers a fingerprint of the molecular structure of the precursor, and, as long as it can be reproducibly acquired, it can be used to identify ionised molecules and fragment ions.^[^^6^^]^

The separation of metabolites prior to detection is often achieved used liquid chromatography (LC) or capillary electrophoresis (CE). Ionisation is mostly achieved through soft ionisation techniques like, e.g., electrospray ionisation (ESI). The ions generated in the ESI source can be fragmented using CID. Regrettably, although the CID spectra are rich in information, it remains difficult to acquire data in a reproducible manner.^[^^7^^,^ ^8^^]^

This is mainly due to the fact that, in beam-type instruments, the precursor ion’s internal energy is difficult to control. More reproducible fragmentation spectra can be produced using ion traps,^[^^9^^]^ which require collisional cooling of the precursor ion for efficient trapping and selective (resonance) excitation. Furthermore, by using multistage MS (MS^n^) experiments, ion trap instruments can provide detailed information on the fragmentation, thereby helping to characterise the structures of metabolites.

Despite the growing popularity of versatile ion trap instruments, in-depth analysis of MS^n^ spectra remains difficult due to the lack of generic software tools. The challenge stems from the multidimensionality of MS^n^ data. The majority of the MS analysis software is well suited for analysing spectra, but not for analysing one of the most important features of MS^n^ data: the precursor-product relations between the ions observed in separate MS^n^ spectra. The only software available at the moment which can be used to analyse and/or compare MS^n^ spectra is Mass Frontier (HighChem, Bratislava, Slovakia).^[^^10^^]^ This proprietary software package, being not open-source, cannot be easily integrated into our specific workflow because it is designed to work only with the propriety data format of one vendor. Furthermore, we wanted to remove spectral artefacts using the hierarchy of observed fragments. This would require software tools that can exchange data using common mass spectrometric data exchange formats such as mzXML,^[^^11^^]^ mzData^[^^12^^]^ or mzML.^[^^13^^]^ As a result, we decided to develop the necessary software ourselves. Our software package, called Multistage Elemental Formula generator (MEF),^[^^14^^]^ used the precursor-product ion relations in order to effectively and specifically extract the relevant data from multistage mass spectra.

Approaches to metabolite identification that use MS^n^ fragmentation often require manual intervention by mass spectrometry experts.^[^^9^^,^^15^^–^^17^^]^ Recently, more automated approaches are reported that greatly facilitate this tedious analysis.^[^^14^^,^^18^^–^^21^^]^ Some of the methods focus on predicting fragmentation patterns *in silico*.^[^^19^^–^^22^^]^ In contrast to these approaches we do not predict the hierarchy of the fragmentation trees. Similarly to the approach of Mass Frontier,^[^^10^^]^ the hierarchy is derived from hierarchy of MS^n^ spectra, but the nodes of the fragmentation tree are fragment ions and not the fragmentation spectra as in Mass Frontier. In contrast to the MetFrag approach,^[^^20^^]^ the hierarchy of the ions in our approach is not calculated but observed in hierarchy of MS^n^ spectra. Scheubert *et al*.^[^^19^^]^ applied the method of Rasche *et al.*^[^^22^^]^ to predict MS^n^ spectra and demonstrated that the hierarchy of the fragment ions derived from hierarchy of MS^n^ spectra adds substantially to the model. As more tools become available for MS^n^ analysis and for fragmentation prediction, it is easier to link the MS fragmentation patterns of metabolites to their molecular structure. This, in turn, will greatly facilitate the generic use of MS^n^ spectra in the field of metabolite identification.

Using our MEF tool, elemental formulas were unambiguously assigned to fragment ions.^[^^14^^]^ This tool uses the hierarchy of the fragment ions in an analogous way as previously reported approaches.^[^^19^^,^^22^^,^^23^^]^ Furthermore, the constraints derived from the ions hierarchy allowed us to discard irrelevant artefacts and to efficiently identify the peaks that were relevant for the precursor ion structure. In this way, we were able to store a hierarchical representation of the elemental composition of fragment ions as observed in MS^n^ spectra, together with the data characterising their MS signals, all in the form of a fragmentation tree.

Our final aim is to develop a database-based metabolite identification pipeline which will be reported separately at a later stage. This pipeline is supported by a database filled with the above-described MS^n^ fragmentation patterns. The use of this MS^n^ data, organised in fragmentation trees, will enable facile comparison of obtained results. We aim to use this approach in an on-line or at-line fashion. In such a setup, where a compound with unknown structure elutes from the LC system, one can either generate a fragmentation tree directly on-line or at-line after fraction collection and subsequent infusion through the nano-ESI interface. This fragmentation tree is subsequently evaluated against a database of fragmentation trees of known compounds/structures. Further bioinformatics-based tools, which will be reported separately, will aid in (partial) recognition of fragmentation trees and using an ‘in-house’ developed structure generator, subsequent structure postulation for the unknown. This will complement a metabolite identification pipeline.

However, before being able to assemble such a setup, a robust acquisition and evaluation of MS^n^ data needs to be developed. Here we report on the development of this part of the envisioned pipeline.

We studied the parameters involved in acquiring the fragmentation trees in order to evaluate their robustness and reproducibility. In addition, we evaluate how these factors affect the topology of the resulting fragmentation tree. Furthermore, we assess the possibility of using this approach as well to discern between structurally related isomeric structures. For the latter we studied two isomeric prostaglandins and several eicosanoids.

## EXPERIMENTAL

### Materials and samples

Glutathione was purchased from Sigma-Aldrich (Steinheim, Germany). Prostaglandin D2 and E2 and all other eicosanoids were obtained from Cayman Chemicals (Ann Arbor, MI, USA). All the samples were dissolved in 50% methanol/0.1% formic acid prior to acquisition. Samples were spun down (5 min, 15 000 *g*) before being transferred into the 96-well sample plate of the NanoMate to prevent clogging of the nano-spray emitter by small particulate matter. Methanol (absolute, ULC/MS grade), water (ULC/MS grade) and formic acid (99%, LC/MS grade) were obtained from Biosolve BV (Valkenswaard, The Netherlands).

### Mass spectrometry

Single (MS) and tandem mass spectrometry (MS/MS) experiments were performed on an LTQ-Orbitrap XL mass spectrometer (Thermo Fisher Scientific, Waltham, MA) controlled by Xcalibur software (version 2.0.7). The instrument was equipped with a TriVersa NanoMate (Advion, Ithaca, NY, USA) nano-electrospray ionisation source. For the positive ionisation mode, the nitrogen pressure was set at 0.45 psi and the ESI voltage was 1.35 kV; for the negative ionisation mode the settings were 0.7 psi and 1.05 kV, respectively. The distance between the ESI chip and the capillary was approximately 5 mm. The MS method was programmed in Xcalibur and consisted of 107 scan events: one full scan and 106 data-dependent tandem mass spectrometry scans up to MS^5^. The method allowed for the fragmentation of the five highest peaks of the MS^2^ and MS^3^ spectra and the three highest peaks of the MS^4^ spectra (this method, with some minor modifications, was successfully demonstrated and reported on earlier^[^^9^^]^). All the spectra acquired were spectra combined from 3 µscans. Full scan spectra (from 50 *m/z* units below to 50 *m/z* units above the molecular weight of the compound in question) were acquired with a resolving power of 30 000 (FWHM at *m/z* 400). In the case of the MS^2–5^ spectra, this resolving power was reduced to 15 000 (FWHM specified at 400 *m/z*) to speed up acquisition. Automatic gain control (AGC) was active at default settings. The fragmentation spectra were acquired for singly charged ions with a precursor intensity threshold of 4500 ion counts. The isolation width for isolating the precursor ion varied from 1 to 3 *m/z* units and we used a normalised collision energy of 25 to 45%. Each measurement was performed in duplicate and within a measurement the MS^n^ sequence was repeated at least 5 times within 15 min.

**Figure 1:**
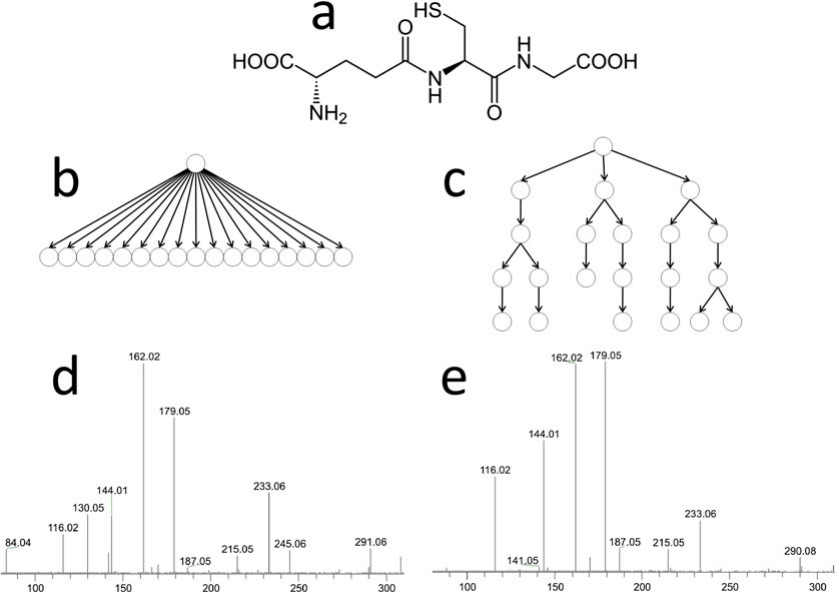
Comparison of MS/MS and MS^n^ fragmentation spectra of glutathione. Schematic hierarchical representation of precursor-product relationships between ions derived from hierarchy of spectra in (b) MS/MS and (c) MS^n^ of glutathione(CHEBI:16856) (a). The corresponding spectra are (d) MS/MS spectrum and (e) total composite spectrum of MS^n^ experiment. The major differences between the two spectra lie in the different signal intensity ratio; most of the fragments (except 84 *m/z*) were observed in both spectra.

The glutathione reference fragmentation spectrum (Fig. 1) was acquired using the HCD cell on the LTQ-Orbitrap XL.

### Data processing

Orbitrap MS^n^ spectra were converted from Thermo’s Xcalibur’s own acquisition (*.RAW) files into mzXML format^[^^11^^]^ using the ReAdW (version 4.3.1) conversion tool provided by the Institute for Systems Biology (Seattle, WA, USA).^[^^24^^]^ mzXML was chosen because it is open and it preserves the precursor mass attributes that point to the hierarchy of fragmentation spectra. Subsequently, mzXML files were analysed using XCMS software^[^^25^^]^ in order to select mass peaks without losing the hierarchical relations between them. We used default XCMS settings for peak picking: the mzGap was 0.2 *m/z* and the signal-to-noise ratio was 10. At this step most of the noise was removed (see Supporting Information on the noise removal). Each mass peak in the resulting table was assigned a precursor ion. We then used the Multi-stage Elemental Formula (MEF) generator^[^^14^^,^^26^^]^ to unambiguously assign elemental formulas to fragment ions and to neutral losses (with 6 ppm mass tolerance), as well as to remove spectral artefacts. In the process of elemental formula assignment we allowed CHNOPS elements and we restricted number and ratio of allowed elements following the rules published by Kind and Fiehn.^[^^5^^]^ MS^1^ isotope pattern information was not used for assigning the precursor ion and fragments. The assigned elemental formulas were constrained by non-integer RDBE (Ring Double Bond Equivalents). This assignment was performed separately for each within-file repetition of the MS^n^ sequence. The resulting fragmentation trees were compared and the peaks that were not present in at least 40% of the trees were discarded. The results were stored in a Chemical Markup Language^[^^27^^]^ (CML) format which combines mass spectrometric and chemical information in a single exchange file. Each step of the analysis, as well as a detailed explanation of the MEF algorithm, can be found in Rojas-Cherto *et al*.^[^^14^^]^ and the application of the algorithm and details of data processing in Rojas-Cherto *et al*.^[^^28^^]^ The detailed list of parameters is provided in the Supporting Information.

In order to distinguish the isomeric prostaglandins, we performed a hierarchical clustering analysis using the R software environment.^[^^29^^]^ The fragmentation trees were represented as vectors of occurrences of elemental formula paths (EFPs), and Euclidean distance was used as a similarity measure in mean linkage clustering. For clustering, we used the complete-link bottom-up algorithm.^[^^30^^]^

The similarity measure of the pairs of isomeric molecules was calculated by applying the Tanimoto coefficient^[^^31^^]^ using the CDK 2D-fingerprint library.^[^^32^^]^ The Tanimoto coefficient was calculated by dividing the number of common EFPs observed for both metabolites by the total number of unique EFPs present for each metabolite minus the number EFPs present in both molecules.

To demonstrate the capability of experimental setup and to evaluate the specificity of analysis a dot-product comparison^[^^33^^]^ of composite spectra of eisosanoids was performed (see Supporting Information).

## RESULTS AND DISCUSSION

### Acquisition of the fragmentation trees

**Figure 2:**
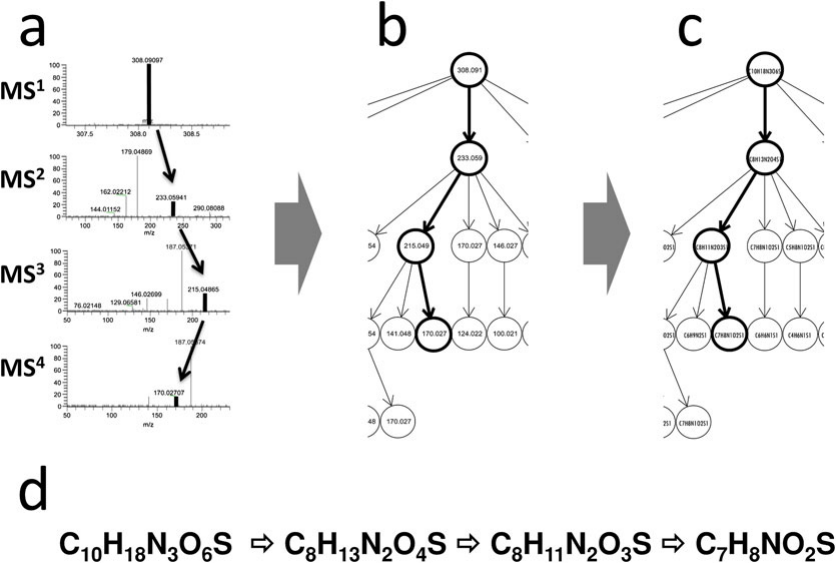
Schematic workflow of fragmentation tree creation shown on example of single elemental formula path. Peaks and their precursor-product relationships depicted in (b) were extracted from MS^n^ spectra (a). Multistage Elemental Formula generator software was used to create a fragmentation tree (c). At this stage, we discarded peaks that were assigned an elemental formula that did not fit in the fragmentation tree. To facilitate unambiguous identification of the fragment ions within the tree they were described by an/their elemental formula path (EFP). The elemental formula path (EFP) (d) unambiguously identifies the fragment ion within the obtained fragmentation tree. The corresponding peaks, masses and elemental formulas are depicted in bold.

The MS^n^ experiments were performed using an LTQ-Orbitrap mass spectrometer. Since this instrument has a high dynamic range in terms of mass accuracy, it allows assignment of elemental formulas both to precursor ions and to their fragment ions. Since fragmentation is performed in a linear ion trap, the high yield for fragment ions (MS/MS efficiency) and the fast duty cycle facilitates extensive MS^n^ experiments in a relatively short period of time.^[^^34^^]^ Moreover, due to the selective resonance excitation of the precursor ion in the ion trap,^[^^35^^]^ the fragment ions obtained do not fragment and can be used as precursor ions for the next stage in the MS^n^ experiment. In this manner, the hierarchy of the MS^n^ spectra determines the hierarchy of the fragment ions (as shown in Figs. 1(c) and 2).

To efficiently extract the hierarchy of these fragment ions, we used our own software, i.e. the Multi-stage Elemental Formula (MEF) generator.^[^^14^^]^ The precursor-product ion relationships between all the mass peaks in the MS^n^ spectra (Figs. 2(a) and 2(b)) were used as constraints in assigning elemental formulas to the individual fragment ions (Fig. 2(c)). Specifically, the elemental formula of a fragment ion cannot contain more atoms of a certain element than its precursor ion, and a precursor ion cannot contain fewer atoms of an element than its fragment. Finally, the assigned elemental formula of a neutral loss and the elemental formula of the fragment have to add up to the elemental formula of the precursor.^[^^14^^]^ In order to unambiguously identify the hierarchical relationship between precursor and product ions, we generated an Elemental Formula Path (EFP) for each ion in the fragmentation tree (see Table 1). An EFP is a list of elemental formulas assigned to consecutive precursor and product ions leading to a particular fragment ion (Fig. 2(d)). In this way, a fragmentation tree can be represented as a collection of EFPs. Consequently, comparing the MS^n^ results of various compounds no longer requires a direct comparison between single fragmentation spectra of specific precursor ions, but one can compare individual fragmentation trees to see whether they include a particular EFP or a set of EFPs (Fig. 2(d)).

In the process of assigning these elemental formulas, we discard the mass peaks that fail to satisfy the constraints derived from the hierarchical precursor-product relations. As a result, artefact peaks not satisfying the above-mentioned constraints, and originating from radio-frequency interference, electronic noise, or the side bands often observed in FT-MS systems^[^^36^^]^ are rejected. How noise and artefacts are removed during the data analysis was described in more detail by Rojas-Cherto *et al*.^[^^14^^,^^26^^]^

We optimised the MS^n^ acquisition protocol so as to yield fragmentation trees that consist of as many fragments as possible, and that can (easily) be reproduced. Since fragments convey structurally relevant information, it is better to have exhaustive ‘wide and deep’ fragmentation trees.

In our approach we optimise spraying conditions, e.g. electrospray emitter voltage, solvent (see Experimental), temperature, etc., to obtain predominantly (de-)protonated molecules, depending on the mode of operation (positive ionisation (PI) or negative ionisation (NI)). These ions are subsequently used as precursor ions for fragmentation tree generation. In practice, however, one will observe a variety of adducts or source fragments in (nano-)ESI, e.g. [M + Na]^+^, [M + NH_4_]^+^, [M + H–H_2_O]^+^ in the case of PI mode or an [M + formate]^–^, [M + acetate]^–^ ion the case of NI mode of operation.^[^^37^^–^^39^^]^ In some cases even, only ions are observed in either the PI or the NI mode. In these cases, only the obtained tree will be stored. The nature of the ESI spectrum depends on e.g. the structure of the compound, and its proton affinity or gas-phase acidity, as well as the solvent composition and pH. Especially when compounds are eluted from an LC system into a nano-ESI source, adducts tend to be formed. However, in most cases (i) many of these adducts do produce after the 1^st^ fragmentation step a (de-)protonated molecule, (ii) the (de-)protonated molecule is mostly present next to the observed adducts, and (iii) the presence of this (de-)protonated molecule is less influenced by actual spraying conditions in contrast to e.g. the [M + Na]^+^ ion. Therefore, our initial approach focuses around the fragmentation of the [M + H]^+^ or the [M–H]^–^ ion. In a later stage we will evaluate how to incorporate fragmentation trees from initial precursor ions not being a [M + H]^+^ or a [M–H]^–^ ion.

The fragmentation of ions strongly depends on mass spectrometric conditions, and these can be controlled using a number of parameters. Some parameters, such as resolution and isolation width, do not directly control the fragmentation process, but instead influence the detection and selection of ions, while other parameters do control the fragmentation process, such as collision energy, activation Q and activation time. Keeping other parameters at default settings, we compared fragmentation trees with different values for isolation width and collision energy, in order to assess the influence of these two parameters.

In order to facilitate the analysis of MS^n^ spectra, the isolation width was adjusted so that we could, on the one hand, optimise sensitivity, and on the other hand isolate the monoisotopic peak of the precursor ion without also isolating its ^13^C isotopic peak. The absence of isotopic peaks in the fragmentation spectra prevented us from co-isolating fragment ions in subsequent MS^n^ stages, because their monoisotopic peaks were always at least 1 *m/z* unit apart (in singly charged ions).

**Figure 3:**
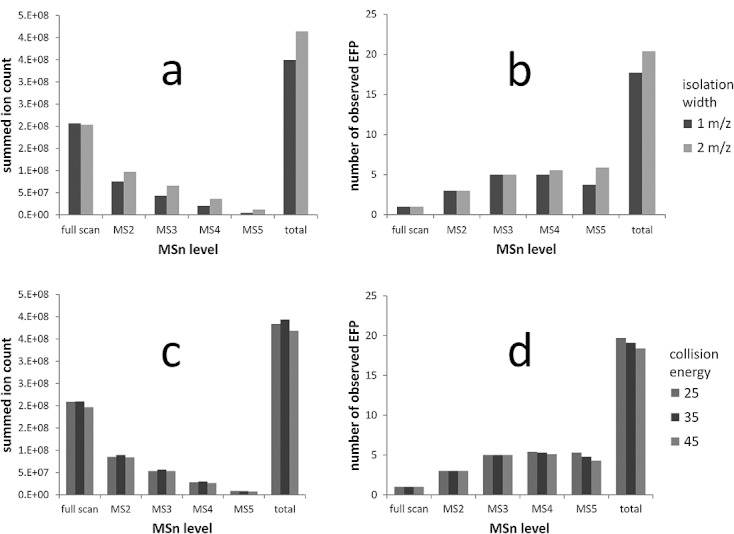
Influence of isolation width on summed ion count (a) and number of detected EFP (b), and influence of collision energy on summed ion count (c) and number of detected EFP (d) in MS^n^ spectra obtained for glutathione.

How these acquisition parameters (width of precursor ion isolation window and normalised collision energy) affected the detection of fragment ions is illustrated in Fig. 3. A number of conclusions can be drawn from this figure. The first observation is that, as expected, the total ion count of the fragments observed in various MS^n^ stages decreases as the number of MS stages increases (Figs. 3(a) and 3(c)). This is caused by a number of factors such as (i) the loss of ions during the trapping, isolation and activation of both precursor and fragments ions in the different stages of the MS^n^ experiments, (ii) the fact that ions below 1/3 of the precursor ion *m/z* ratio are not trapped in the ion trap, and (iii) the fact that ions below *m/z* 50 cannot be detected using the Orbitrap detector. Since we were not investigating the relative importance of these factors in this study, we can merely observe that the total ion intensity diminished as a result of multiple MS^n^ levels.

The second observation is that, on average, an isolation width of 2 *m/z* (M ± 1 *m/z*) units led to higher total ion counts than an isolation width of 1 *m/z* (M ± 0.5 *m/z*) unit (Fig. 3(a)). This held for all the fragments observed. As a consequence of the higher peak intensities, the total number of EFPs detected in the fragmentation trees was, on average, 15% higher when using a wider isolation width (Fig. 3(b)). Thirdly, collision energy only had a marginal effect on the overall intensity (Fig. 3(c)) and on the number of observed fragment ions and EFPs (Fig. 3(d)).

**Figure 4:**
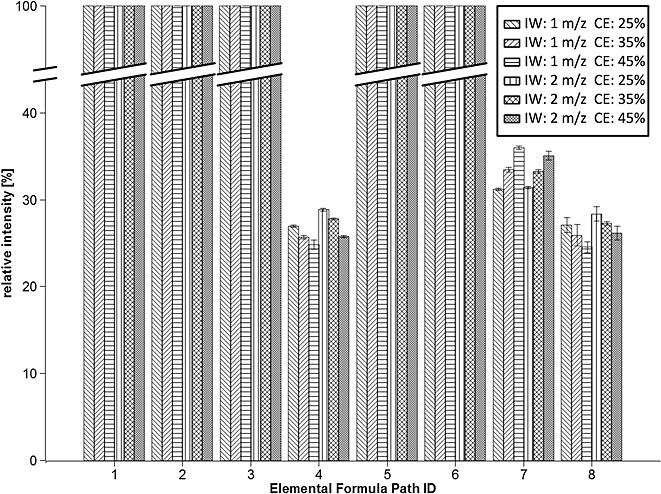
Comparison of fragmentation trees acquired using various mass isolation widths (IW, 1 *m/z* and 2*m/z*) and normalised collision energy (CE, 25%, 35%, 45%) (shown on representative elemental formula paths from each level of MS^n^ spectra for clarity (see Table 1 for identity of elemental formula paths). The relative intensity within a spectrum of according mass peaks is plotted with standard deviation.

A more detailed comparison of the effects of these acquisition parameters on the resulting fragmentation trees is shown in Fig. 4. This figure plots the effect of the tested acquisition parameters on the relative intensities for particular EFPs. Clearly, the isolation width parameter did not significantly impact the relative intensity of the fragment ion peaks. Collision energy, on the other hand, did, as expected, influence these intensities, and consequently the ratio between fragment ions, although its influence turned out to be minor. In addition, we observed a small standard deviation of the relative intensity of the fragment ions (typically less than 2%, and max. 9%). Basically, all tested collision energy settings yielded highly similar spectra.

**Table I:** List of elemental formula paths constituting the fragmentation tree of glutathione

ID	Elemental Formula Path	MS^n^	Elemental Formula	Mass	Relative intensity (%)	Mass error [ppm]	Standard deviation relative intensity
1	C10H18N3O6S1	1	C10H18N3O6S	308.091	100	−0.5	0
2	C10H18N3O6S1||C5H11N2O3S1	2	C5H11N2O3S	179.049	100	1.3	0
3	C10H18N3O6S1||C5H11N2O3S1||C5H8N1O3S1	3	C5H8NO3S	162.022	100	0.2	0
4	C10H18N3O6S1||C5H11N2O3S1||C5H8N1O3S1||C4H6N1O1S1	4	C4H6NOS	116.016	27	−0.3	1.4
5	C10H18N3O6S1||C5H11N2O3S1||C5H8N1O3S1||C5H6N1O2S1	4	C5H6NO2S	144.011	100	0.1	0
6	C10H18N3O6S1||C5H11N2O3S1||C5H8N1O3S1||C5H6N1O2S1||C4H6N1O1S1	5	C4H6NOS	116.016	100	−0.4	0
7	C10H18N3O6S1||C5H8N1O3S1	2	C5H8NO3S	162.022	33	1.3	1.8
8	C10H18N3O6S1||C5H8N1O3S1||C4H6N1O1S1	3	C4H6NOS	116.016	27	−0.4	1.4
9	C10H18N3O6S1||C5H8N1O3S1||C5H6N1O2S1	3	C5H6NO2S	144.011	100	0.1	0
10	C10H18N3O6S1||C5H8N1O3S1||C5H6N1O2S1||C4H6N1O1S1	4	C4H6NOS	116.016	100	−0.5	0
11	C10H18N3O6S1||C8H13N2O4S1	2	C8H13N2O4S	233.059	25	1.7	1.4
12	C10H18N3O6S1||C8H13N2O4S1||C7H11N2O2S1	3	C7H11N2O2S	187.054	100	0.8	0
13	C10H18N3O6S1||C8H13N2O4S1||C7H11N2O2S1||C7H8N1O2S1	4	C7H8NO2S	170.027	100	0.5	0
14	C10H18N3O6S1||C8H13N2O4S1||C7H11N2O2S1||C7H8N1O2S1||C6H6N1S1	5	C6H6NS	124.022	100	−0.4	0
15	C10H18N3O6S1||C8H13N2O4S1||C8H11N2O3S1	3	C8H11N2O3S	215.049	32	0.9	3.5
16	C10H18N3O6S1||C8H13N2O4S1||C8H11N2O3S1||C7H11N2O2S1	4	C7H11N2O2S	187.054	100	0.8	0
17	C10H18N3O6S1||C8H13N2O4S1||C8H11N2O3S1||C7H11N2O2S1||C6H9N2S1	5	C6H9N2S	141.048	100	−0.1	0
18	C10H18N3O6S1||C8H13N2O4S1||C8H11N2O3S1||C7H11N2O2S1||C7H8N1O2S1	5	C7H8NO2S	170.027	82	0.3	8.7
19	C10H18N3O6S1||C5H11N2O3S1||C5H8N1O3S1||C4H6N1O1S1||C3H6N1S1	5	C3H6NS	88.021	100	−1.0	0
20	C10H18N3O6S1||C5H8N1O3S1||C4H6N1O1S1||C3H6N1S1	4	C3H6NS	88.021	100	−1.0	0
21	C10H18N3O6S1||C5H8N1O3S1||C5H6N1O2S1||C4H6N1O1S1||C3H6N1S1	5	C3H6NS	88.021	100	−1.0	0

### Reproducibility and robustness – the effects of the concentration of the analyte on the size and shape of the fragmentation tree

**Figure 5:**
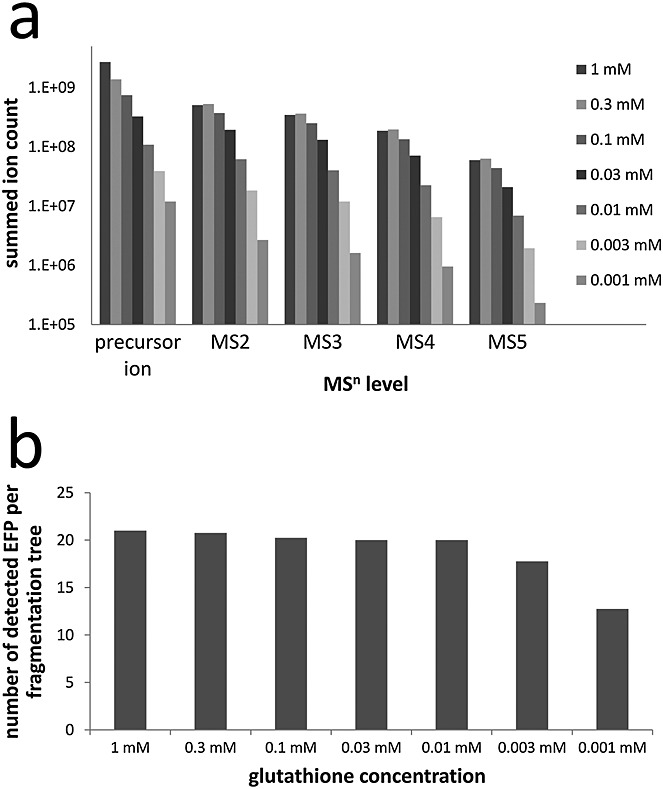
Influence of glutathione concentration on (a) the sum ion count of the mass peaks detected in its fragmentation tree (the ion count is plotted on logarithmic scale) and (b) the number of elemental formula paths detected in the fragmentation tree of glutathione.

In order to study the influence of the analyte concentration on the reproducibility and robustness of the obtained fragmentation tree, trees were generated from MS^n^ spectra acquired with various concentrations of glutathione (ranging from 1 μM to 1 mM). The absolute intensities of fragment ions are directly related to the absolute intensity of their precursor ion, which in turn depends on the concentration of the analyte. The effect of the concentration of glutathione on the abundance of the observed fragment ions is illustrated in Fig. 5(a). As can be seen, the total summed intensity of the fragment ions in all MS^n^ levels was influenced by the absolute intensity of the precursor ion (MS^1^), with one exception: fragment intensities for the two highest concentrations (1 mM and 0.3 mM) seem to be the same even though the intensity of precursor ions differs. This is probably due to the fact that the number of charges which can be retained in the ion trap is limited. The analyte concentration also influenced both the total size of the obtained fragmentation tree and the total number of fragments detected in a tree, as can be seen in Fig. 5(b). From 21 elemental formula paths observed for 1 mM glutathione, 20 were observed for all glutathione concentrations in the range from 10 μM to 1 mM. Only the two lowest concentrations tested (3 μM and 1 μM) yielded smaller fragmentation trees, consisting respectively of 18 and 13 EFPs. The number of detected ions was most reduced in MS^4^ and MS^5^ spectra (reduction by almost 50%), but it was also reduced in MS^3^ and MS^2^ spectra. Clearly, for all higher concentrations the instrument was able to compensate for the lower ion abundances by longer ion accumulation times, yielding fragment-rich MS^n^ spectra. Moreover, a comparison of the relative peak intensity obtained for each detected EFP demonstrated that the ratios between peak abundances can easily be reproduced across the whole range of glutathione concentrations (data not shown). As expected, the largest deviation was observed for low intensity peaks, which were characterised by the lowest signal-to-noise ratio. The results show that fragmentation tree topology can be acquired in a robust way and the analyte concentration has a minor effect on the overall arrangement of the fragmentation tree.

### Specificity of analysis – fragmentation tree structure characteristic for isomeric prostaglandins

Isomerism is commonly observed in metabolites and specific isomers often have a unique biological function in a living organism. Therefore, a successful metabolite identification method, selective enough to discern between isomeric structures, is essential for understanding the biochemical roles of each individual isomer. Although tandem mass spectrometry inherently cannot distinguish enantiomers^[^^40^^]^ – for that we might consider derivatisation with a chiral label or separation with chiral chromatography prior to MS analysis – it can, in principle, differentiate between individual constitutional isomers and/or diastereoisomers.^[^^41^^]^

**Figure 6:**
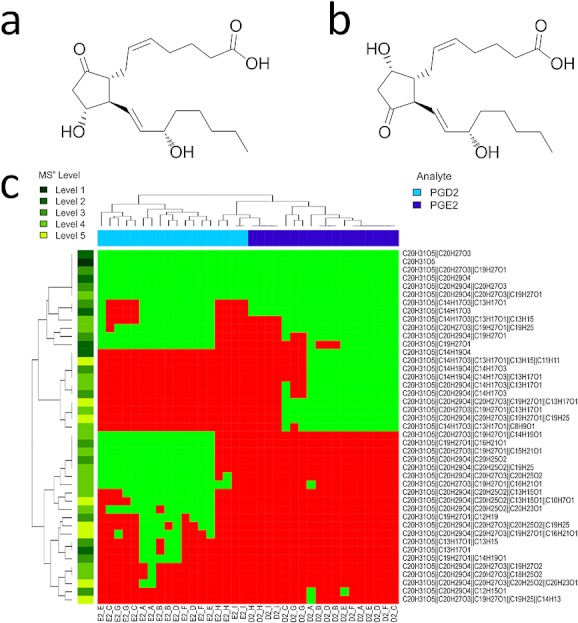
Chemical structures of prostaglandin E2 (CHEBI:15551) (a) and D2 (CHEBI:15555) (b) and clustered heatmap analysis of fragmentation trees acquired from various concentrations of prostaglandin E2 and prostaglandin D2 (green blocks denote detected, red blocks undetected EFPs). Fragmentation trees were acquired in duplicate from each concentration (denoted with letters A–I: A – 100 μM, B – 30 μM, C – 10 μM, D – 3 μM, E – 1 μM, F – 0.3 μM, G – 0.1 μM, H – 0.03 μM, I – 0.01 μM). Both prostaglandins form separate clusters due to the observed characteristic EFPs, except in the case of the two lowest concentrations (H and I), where we observed no characteristic EFPs.

In order to assess the feasibility of discerning between isomers on the basis of an analysis of their fragmentation trees, the above-mentioned protocol was used on two isomeric prostaglandins: D2 and E2 (Figs. 6(a) and 6(b), respectively). Prostaglandins are important mediators of (patho)-physiological effects.^[^^42^^]^ Although chemically very similar, prostaglandin D2 (PGD2) and prostaglandin E2 (PGE2) have different biological functions. It is challenging but crucial for studies on biological systems to be able to distinguish such closely related structures reliably in a wide range of concentrations.

We checked the repeatability and robustness of the acquisition of a fragmentation tree for both prostaglandins over a range of concentrations, *viz*. from 10 nM to 100 μM, in the NI mode. This polarity was used because the protonated molecule is not observed in PI mode due to predominant water loss.^[^^43^^]^ Since, as demonstrated above for glutathione, the analyte concentration influences the size of the resulting fragmentation tree, it was important in the case of prostaglandins to establish whether their concentration and the size of the resulting fragmentation trees interfered with the analysis aimed at distinguishing the two isomers.

The analysis of the obtained fragmentation trees shows that both prostaglandins yield a similar number of fragment masses in their fragmentation trees (14 for PGD2 and 18 for PGE2). These fragments constitute 25 EFPs in the fragmentation tree of PGD2 and 31 EFPs in the tree of PGE2 (Fig. 6(c)). Despite their structural similarity, only 13 elemental formula paths are detected for both prostaglandins; 12 of the observed elemental formula paths are characteristic for PGD2 and 18 for PGE2. In total, 43 unique elemental formula paths are detected, consisting of 32 unique elemental formulas of fragment ions. Because the number of EFPs that we detect is higher than the number of observed fragment masses (as some fragment masses were observed in more than one spectrum), we postulate that the number of characteristic features for each prostaglandin is higher than the number of characteristic features (peaks) that were reported in tandem mass spectra.^[^^44^^]^ This means that our method is more specific than a method relying on tandem mass spectra.

Comparing the fragmentation trees obtained from various concentrations of prostaglandins reveals that lowering the concentration influences the tree topology while preserving the characteristic elemental formula paths which distinguish between the isomers. Obviously, the fragmentation trees obtained from higher concentrations consist of more EFPs than the fragmentation trees from lower concentrations (Fig. 6(c)). In addition, as can be seen, the number of observed characteristic EFPs for each prostaglandin decreases with the concentration.

The differences between the fragmentation trees of PGD2 and PGE2 acquired from various concentrations are visualised in a clustered heatmap (Fig. 6(c)). As a result of the above-mentioned detection of characteristic EFPs, the fragmentation trees of the two prostaglandins form separate clusters. In the case of the two lowest concentrations (0.03 μM and 0.01 μM (H and I, respectively)) we only observe EFPs that are common to the two prostaglandins. These results suggest that with concentrations of 0.1 μM and higher, isomeric prostaglandins can be distinguished unambiguously. Furthermore, it shows that minor fluctuations in the topology of a fragmentation tree between repetitions are negligibly smaller compared to the differences observed between isomeric metabolites, which proves the selectivity of our approach.

### Specificity of analysis – distinguishing isomers

**Figure 7:**
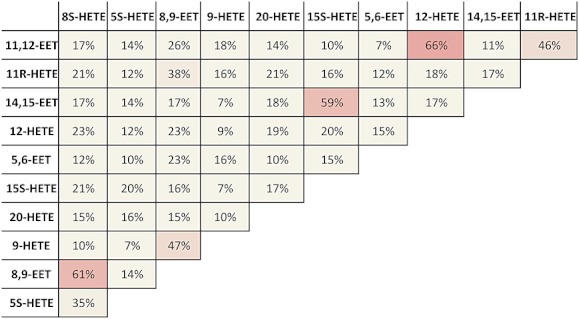
Similarity of the fragmentation trees of isomeric eicosanoids given as Tanimoto coefficient. Eleven eicosanoids (see Table 2) constitute 55 pairs of isomers. The similarity lower than 30% allows for unambiguous distinguishing of fragmentation trees.

The approach demonstrated above on the pair of isomeric prostaglandins was evaluated on a set of 11 isomeric eicosanoids (Table 2), constituting 55 pairs of isomers. The similarity of fragmentation trees within each pair was given as a Tanimoto coefficient^[^^31^^]^ (see Fig. 7). The identical analysis of the fragmentation trees of isomeric prostaglandins (Fig. 6) yielded Tanimoto coefficients of more than 70% for replicate measurements, more than 60% for similar fragmentation trees, and less than 30% for dissimilar fragmentation trees (data not shown). These findings are a good indication for the interpretation of the data on the above-mentioned eicosanoids. It can be seen in Fig. 7 that for the majority of fragmentation tree pairs the isomers can be distinguished: 48 pairs out of 55 yielded Tanimoto coefficients lower than 30%. The remaining 7 pairs yielded Tanimoto coefficients lower than 70% implying dissimilarity. Furthermore, comparing fragmentation trees using this approach gave sharper distinction between isomers than dot-product comparison of composite spectra (Supplementary Fig. S1, see Supporting Information). These results support the conclusion that using fragmentation trees as in our approach results in differentiation of positional isomers.

**Table 2:** List of 11 isomeric eicosanoids

ID	Name	Lipid maps ID
8S-HETE	8*S*-hydroxy-5*Z*,9*E*,11*Z*,14*Z*-eicosatetraenoic acid	LMFA03060006
5S-HETE	5*S*-hydroxy-6*E*,8*Z*,11*Z*,14*Z*-eicosatetraenoic acid	LMFA03060002
8,9-EET	8,9-epoxy-5*Z*,11*Z*,14*Z*-eicosatrienoic acid	LMFA03080003
9-HETE	9-hydroxy-5*Z*,7*E*,11*Z*,14*Z*-eicosatetraenoic acid	LMFA03060089
20-HETE	20-hydroxy-5*Z*,8*Z*,11*Z*,14*Z*-eicosatetraenoic acid	LMFA03060009
15S-HETE	15*S*-hydroxy-5*Z*,8*Z*,11*Z*,13*E*-eicosatetraenoic acid	LMFA03060001
5,6-EET	5,6-epoxy-8*Z*,11*Z*,14*Z*-eicosatrienoic acid	LMFA03080002
12-HETE	12-hydroxy-5*Z*,8*Z*,10*E*,14*Z*-eicosatetraenoic acid	LMFA03060088
14,15-EET	14,15-epoxy-5*Z*,8*Z*,11*Z*-eicosatrienoic acid	LMFA03080005
11R-HETE	11*R*-hydroxy-5*Z*,8*Z*,12*E*,14*Z*-eicosatetraenoic acid	LMFA03060028
11,12-EET	11,12-epoxy-5*Z*,8*Z*,14*Z*-eicosatrienoic acid	LMFA03080004

Especially in the case of isomeric structures one can envision that having actual annotations of elemental formulae of fragment ions, neutral losses and as well chemical structures of fragment ions in the database would be highly desirable. In order to accomplish this we store fragmentation trees consisting of mass spectrometric data annotated with elemental formula of fragment ions and neutral losses, using Chemical Markup Language (CML). CML supports various chemical concepts, such as reactions and molecules, and makes it possible to store the chemical structure of each ion in InChi format. To assist further structure elucidation, structural annotation of these fragmentation trees will be added in a later stage. Manual annotation of a fragmentation tree collection could be developed by experts and the mass spectrometric society as an open project. Information on the actual structures, or the most likely structures, will allow for (sub-)structure recognition and immensely assist in the identification endeavour of unknowns. This will hugely impact the way in which we interpret fragmentation spectra.

## CONCLUSIONS

Multistage mass spectrometry has, due to the popularity of ion trap instruments, become a very powerful technique for structural characterisation in e.g. metabolomics. Although the technique is well known, until now the resulting MS^n^ data could not be straightforwardly analysed, and the results were only accessible as collections of related fragmentation spectra. We demonstrate that the use of constraints derived from the precursor-product ion relationships of the ions observed in MS^n^ spectra not only allows to efficiently remove artefacts, it also allows us to assign unambiguously, elemental formula to each relevant fragment ion. Reproducibly representing MS^n^ spectra as fragmentation trees allows for facile comparison of the fragmentation data of individual metabolites. This is extremely beneficial in view of our envisioned database-based metabolite identification pipeline. Although we only demonstrate this approach by means of data of several compounds, fragmentation trees (PI and NI in most cases) of approx. 500 individual compounds (including ca. 100 isomers) present in human biofluids have in the meantime been acquired and are being evaluated. These compounds belong to a wide set of compound classes and span a large part of the (human) metabolome.

The adoption of this approach will greatly depend on accessibility of public databases which store and exchange annotated fragmentation tree data. The data format used must accommodate both mass spectrometric and chemical data. To our knowledge, the only data format fulfilling this requirement is Chemical Markup Language (CML).^[^^27^^]^ The reproducibility and robustness of the acquisition of fragmentation trees suggests that they can potentially be used in computer-aided generic metabolite identification methods. However, in order to fully evaluate the feasibility of this approach, between-lab reproducibility must be assessed.
