# Supplementary material for: Fragmentation trees for the structural characterisation of metabolites
Source: Rapid Commun Mass Spectrom. 2012 Aug 31;26(19):2275–86. doi: 10.1002/rcm.6340 (PMC3573646; doi:10.1002/rcm.6340)
Supplement: Supplementary file 1 [file rcm0026-2275-sd1.doc]

**SUPPORTING INFORMATION**

**Fragmentation trees for the structural characterisation of metabolites**

**Piotr T. Kasper^1,2^, Miguel Rojas-Chertó^1,2^, Robert Mistrik^3^, Theo Reijmers^1,2^, Thomas Hankemeier^1,2^ and Rob J. Vreeken^1,2^**

^1^Netherlands Metabolomics Centre, Einsteinweg 55, Leiden, The Netherlands

^2^Leiden/Amsterdam Centre for Drug Research (LACDR), Leiden University, Einsteinweg 55, Leiden, The Netherlands

^3^HighChem. Ltd., Bratislava, Slovakia

**Correspondence to*: R. J. Vreeken, Netherlands Metabolomics Centre, Einsteinweg 55, Leiden, The Netherlands.

E-mail: r.vreeken@lacdr.leidenuniv.nl

**Library to process and compare MS^n^ data**

The Java library to process and compare MSn data is available as an open source project from Sourceforge at <http://sourceforge.net/projects/samsn>. The README file contains a tutorial explaining the features and the command lines to use.

sn = 10 . Signal to noise threshold

mzgap = 0.2 . Minimal distance between adjacent peaks

rint = 0.0. Relative intensity threshold.

acc = 6 . Mass tolerance in ppm

rules = RDBE . (Ring Double Bond Equivalents) Constraint rules applied to the formula

occurr = 0.4 . Minimum occurrence to appear in all repetitions within one file to be accepted as a fragment.

ec = C0..10,H1..20. Elements to be included, together with the upper-/lower-limit of the number of atoms. They will depend on the compound to be analyzed. E.g. ec=C0..10,H1..20 means that the range of the carbon atom is set between 0 and 10 and the range of the hydrogen atom is set between 1 and 20.

MS^1^ isotope pattern information was not used for assigning the parent ion and fragments.

All MSn data was processed with the above tool using the following command line:

> java -jar sams.jar -occurr=0.4 -sn=10 -mzgap=0.2 -rint=0 -acc=6 -ec=[MY_ELEMENTS] -rules=[RDBER] -imzXML filename.mzXML -ocml filename.cml process

**The noise and artefact removal.**

The noise detection/removing is not a feature of MEF software. After peak picking performed by XCMS (with adjustable signal-to-noise threshold) the remaining noise and artefacts are removed as a result of peak assignment. MEF assigns the set of possible (within the allowed mass tolerance) elemental compositions to each peak and then insures consistence with elemental compositions of the precursor and consequent fragments. The noise is not detected and it is removed by removal of non-relevant peaks (peaks not belonging to the metabolite).

**Dot-product spectra comparison**

To emphasize the capability of the experimental setup the composite spectra of isomers listed in Table 2 were compared using algorithm of dot-product comparison(1). MS^n^ spectra were summed and converted into total composite spectra with Xcalibur version 2.0.7 (Thermo Fisher Scientific, Waltham, MA). These composite spectra were then binned and the dot-product was calculated for each pair of isomers. The results are demonstrated in Figure S1. Although the isomers can be discerned from each other using this approach, the comparison of fragmentation trees using Tanimoto coefficient gives sharper distinction between isomers (Figure 2).


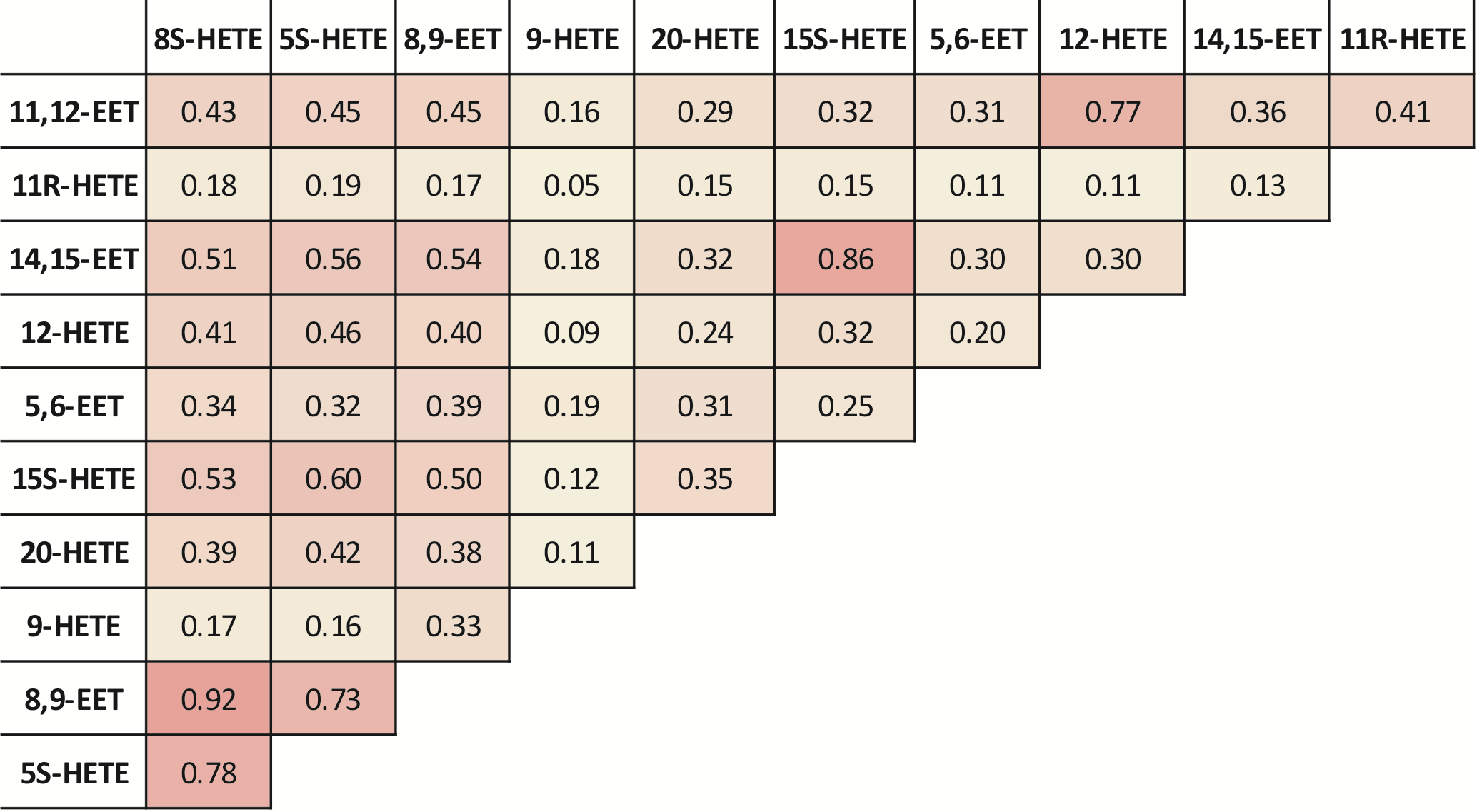


Figure S1. Dot-product comparison of total composite spectra generated from MS^n^ spcectra of 11 eicosanoids (see Table 2) constituting 55 pairs of isomers. See Figure 7 for fragmentation tree comparison.

**The Supporting Data**

The spectra analyzed in this paper (mzXML format) can be downloaded from <http://analyticalbiosciences.leidenuniv.nl/people/kasper>

The MEF software used in the analysis can be downloaded from <http://abs.lacdr.gorlaeus.net/people/rojas-cherto>

1. Stein, S. E., and Scott, D. R. (1994) Optimization and testing of mass spectral library search algorithms for compound identification*, Journal of the American Society for Mass Spectromet*r*y* 5, 859-866.
